# Unraveling the Functional Impact of Splicing Variants in Inherited Hearing Disorders Through Minigene Splicing Assays

**DOI:** 10.3390/biomedicines13092245

**Published:** 2025-09-11

**Authors:** Lara Emily Rosso, Giulia Pianigiani, Anna Morgan, Elisa Rubinato, Elisa Paccagnella, Stefania Lenarduzzi, Anita Wischmeijer, Beatrice Spedicati, Giorgia Girotto

**Affiliations:** 1 Department of Medicine, Surgery and Health Sciences, University of Trieste, 34149 Trieste, Italy; laraemily.rosso@burlo.trieste.it (L.E.R.); elisa.paccagnella@burlo.trieste.it (E.P.); beatrice.spedicati@burlo.trieste.it (B.S.); giorgia.girotto@burlo.trieste.it (G.G.); 2 Institute for Maternal and Child Health—I.R.C.C.S. “Burlo Garofolo”, 34137 Trieste, Italy; anna.morgan@burlo.trieste.it (A.M.); elisa.rubinato@burlo.trieste.it (E.R.); stefania.lenarduzzi@burlo.trieste.it (S.L.); 3 Clinical Genetics Service and South Tyrol Coordination Center for Rare Diseases, Department of Pediatrics, Regional Hospital of Bolzano, 39100 Bolzano, Italy; titiaanita.wischmeijer@sabes.it

**Keywords:** splicing variants, hearing loss, minigene assay

## Abstract

**Background/Objectives**: Hereditary hearing loss (HHL) is a genetically heterogeneous condition, involving more than 150 genes in non-syndromic cases and associated with over 400 distinct disorders in syndromic forms. Although whole-exome sequencing (WES) has markedly increased diagnostic yield, a substantial number of cases remain unsolved, often due to intronic variants that affect splicing and are difficult to interpret. This study aimed to characterize the potential impact of intronic variants predicted to alter splicing in families affected by HHL. **Methods**: The effect of seven intronic variants, previously identified in a diagnostic setting by WES within *ADGRV1*, *ATP11A*, *GSDME*, *OTOF*, *OTOGL*, and *USH2A* genes, was evaluated. To functionally validate these predictions, in vitro minigene splicing assays were subsequently performed. **Results**: All the identified variants were predicted to disrupt normal RNA splicing. The functional studies with minigene assays confirmed this observation and showed that the tested variants induced both exon skipping and activation of cryptic splice sites. In five out of seven cases, these splicing alterations caused a frameshift and introduced a premature termination codon, ultimately resulting in nonsense-mediated mRNA decay and protein degradation. **Conclusions**: This study expands the mutational spectrum of HL-related genes and highlights the importance of integrating in silico predictions with minigene assays. Such a combined approach is crucial for accurate interpretation of splicing variants, particularly when patient-derived RNA samples are unavailable.

## 1. Introduction

Hearing loss (HL) affects over 430 million people worldwide, including 34 million children, and this number is estimated to nearly double by 2050, highlighting a growing public health concern (World Health Organization) [1,2]. Improving diagnostic strategies is crucial for prompt and accurate diagnosis, particularly in cases of hereditary HL (HHL), which accounts for nearly 60% of congenital hearing impairment in developed countries [3]. However, timely diagnosis remains challenging due to the extensive clinical and genetic heterogeneity of HHL [4].

HHL is broadly classified into syndromic (SHL, ~30% of HLL cases) and non-syndromic (NSHL, ~70%) forms. SHL is characterized by hearing impairment accompanied by other clinical features and has been associated with nearly 400 syndromes [5]. For example, in Alport, Pendred, Usher, and Waardenburg syndromes, HL represents the major clinical symptom [5]. NSHL is associated with 156 genes, including 64 autosomal dominant (AD), 88 autosomal recessive (AR), and 7 X-linked (XL) genes, with some of them exhibiting overlapping inheritance patterns (Hereditary Hearing Loss Homepage) [6,7]. Mutations in the *GJB2* gene are the leading cause of autosomal recessive NSHL (ARNSHL) [8,9], followed by variants in the *STRC* gene [10,11,12]. In addition to high genetic heterogeneity, there is wide clinical variability, making diagnosis even more challenging. For both isolated HL and SHL, symptoms may deviate from classical presentations and/or show intrafamilial phenotypic variability. Furthermore, a patient may be initially classified within a non-syndromic condition and subsequently receive a molecular diagnosis for a syndromic condition, due to the different timing of manifestation of associated abnormalities [13]. Additionally, dual molecular diagnoses, involving pathogenic variants in two different genes, may contribute to distinct or overlapping phenotypes [4,14].

The advent of next-generation sequencing (NGS) technologies, including whole-exome sequencing (WES), has dramatically increased the detection rate of HL [15,16,17]. Nevertheless, approximately 50% of cases still remain unsolved [18] as WES covers only 2% of the genome, thus potentially missing pathogenic variants in deep intronic regulatory or non-coding regions that affect splicing [19,20]. Among the different classes of genetic alterations, splicing variants constitute a significant subset, accounting for nearly 9% of reported mutations in the Human Gene Mutation Database (HGMD) [21] and are increasingly recognized as a relevant molecular mechanism underlying the pathogenesis of HL [22,23,24]. Many of these splicing variants are classified as variants of unknown significance (VUS) [20], requiring functional characterization to understand their clinical impact. Several in silico tools have been developed to predict the deleterious effect of genetic variants and their impact on splicing processing, including Combined Annotation Dependent Depletion (CADD) [25], dbscSNV [26], and SpliceAI [27,28]. However, they only provide predictions which should require a functional validation to confirm their biological relevance. To this end, both in vivo and in vitro splicing analyses are fundamental to assess the impact of candidate variants. Although in vivo RNA studies would be optimal, they require readily accessible RNA from disease-relevant tissue, such as the inner ear, which is often not feasible. In addition, given the tissue-specific nature of splicing, analyses on alternative sources, such as blood, may not reflect splicing patterns occurring in the target tissue. To overcome this limitation, the use of in vitro assays is essential. Minigene splicing assays play a key role in assessing the impact of variants on splicing, validating the in silico predictions, and determining how genetic variants alter splicing patterns, potentially leading to abnormal protein production [29]. These functional assays support variant reclassification and improve diagnostic accuracy, especially in SHL cases, with incomplete penetrance or a delayed-onset phenotype. They also have a great impact on genetic counseling, enabling more accurate recurrence risk assessment and guiding reproductive and management decisions.

In this study, we investigated seven splicing variants identified by WES in seven patients affected by both NSHL and SHL. We evaluated the impact of variants on splicing through in silico prediction tools and validated these effects with in vitro minigene splicing assays. Our work aims to clarify the deleterious effect of these variants, ultimately improving the molecular diagnosis of patients with HHL.

## 2. Materials and Methods

### 2.1. Ethical Statement

All the analyses were performed following the relevant guidelines and regulations. Written informed consent was obtained from all participants or, in the case of underage patients, their legal guardians. The study was conducted in accordance with the tenets of the Helsinki Declaration.

### 2.2. Study Participants and Clinical Evaluation

Six HL-affected patients were referred to the Medical Genetics Unit of the I.R.C.C.S. “Burlo Garofolo” (Trieste, Italy) and one to the Clinical Genetics Service and South Tyrol Coordination Center for Rare Diseases of the Regional Hospital of Bolzano (Bolzano, Italy). Subjects underwent a multistep diagnostic approach that included the following steps: (1) detailed clinical examination in order to differentiate NSHL from SHL forms, as previously described [4], (2) direct sequencing of *GJB2* gene and MLPA analysis of *STRC* in NSHL patients, (3) long-range PCR and *STRC* sequencing in patients with a heterozygous *STRC* deletion and an audiometric profile consistent with autosomal recessive deafness type 16 (DFNB16). (4) WES analysis was employed in NSHL patients negative to (2) and (3) tests, and in all SHL patients.

### 2.3. WES Analysis and Interpretation

Genomic DNA (gDNA) was extracted from patients’ peripheral blood using the QIAsymphony Midi Kit (Qiagen, Cat. No. 51183, Venlo, The Netherlands) following the manufacturer’s instructions. DNA quality was analyzed through 1% agarose gel electrophoresis, and its concentration was measured using the Qubit DNA Broad Range kit (Thermo Fisher Scientific, Cat. No. Q33266, Waltham, MA, USA). Trio WES was performed on the Illumina NextSeq 550 platform (Illumina Inc., San Diego, CA, USA) as previously described [4].

### 2.4. In Silico Prediction

The effect of the variants was predicted using CADD v1.7 [25], a computational tool that assesses the deleteriousness of genetic variants, and dbscSNV v1.1 [26], which predicts the impact of variants on RNA splicing. Moreover, the potential effect of identified variants on splicing was assessed by performing an in silico analysis with SpliceAI prediction tool v.1.3.1 (https://spliceailookup.broadinstitute.org, accessed on 1 August 2025) [28,30], a deep neural network that predicts splicing from each position in a pre-mRNA transcript.

### 2.5. Minigene Splicing Assay

Functional validation of selected splicing variants was performed through in vitro minigene splicing assays. Briefly, the exon of interest, along with 150–200 bp of its flanking introns, was PCR-amplified from controls’ and/or patients’ gDNA in both wild-type (wt) and variant-carrying form. PCR amplification was carried out using specific primers (Table 1) and the PrimeSTAR Max DNA Polymerase (Takara Bio USA Inc., Cat. No. R045A, San Jose, CA, USA). The wt amplified fragment and the one harboring the variant were cloned into the XhoI/NotI-digested Exontrap pET01 vector (MoBiTec GmbH, Cat. No. PET01, Göttingen, Germany) using the In-Fusion^®^ Snap Assembly Kit (Takara Bio USA Inc., Cat. No. 060822) according to manufacturer’s instructions. All constructs were next confirmed by Sanger sequencing.

### 2.6. Cell Culture, Transfection, and RNA Extraction

Human Embryonic Kidney 293T cells (HEK293T) were harvested in Dulbecco’s modified Eagle medium (DMEM) with L-glutamin (Euroclone, Cat. No. ECM0728L, Milan, Italy) supplemented with 100 U/mL penicillin, 0.1 mg/mL streptomycin (Euroclone, Cat. No. ECB3001D), and 10% fetal bovine serum (Euroclone, Cat. No. ECS5000L). Cells were grown in mycoplasma-free conditions and maintained at 37 °C with 5% CO_2_. HEK293T cells were seeded into a 6-well plate (0.5 × 10^6^ cells/well) and the day after, once they reached a 70–80% confluence, they were transfected. Transfection was performed using Lipofectamine 2000 Transfection Reagent (Thermo Fisher Scientific, Cat. No. 11668019) and 1.0 μg of wt and variant-carrying plasmids diluted in Opti-MEM Reduced Serum Medium (Thermo Fisher Scientific, Cat. No. 31985062). All minigene transfection experiments were carried out in triplicate.

### 2.7. Reverse Transcription and RT-PCR Analysis

Twenty-four hours after transfection, total RNA was extracted with NucleoSpin RNA Plus kit (Macherey-Nagel, Cat. No. 740984.250, Düren, Germany) and additionally treated with DNase I, Amplification Grade (Thermo Fisher Scientific, Cat. No. 18068015) to remove residual contaminating DNA. RNA concentration and quality were assessed using the Nanodrop One spectrophotometer (Thermo Fisher Scientific, Cat. No. 13-400-518). Reverse transcription was carried out with 500 ng of total RNA and the SensiFAST cDNA Synthesis Kit (Bioline|Meridian Bioscience, Cat. No. BIO-65053, Memphis, TN, USA). PCR analysis was performed with GoTaq DNA Polymerase (Promega, Cat. No. M7132, Madison, WI, USA) and vector-specific primers (Table 1). The cycling conditions were the following: initial denaturation 95 °C–2′, followed by 30 cycles of denaturation 95 °C–30″, annealing 60 °C–30″, extension 72 °C–30″, final extension 72 °C–5′. RT-PCR products were analyzed via electrophoresis on 2% agarose gel and visualized with Midori Green Advance (Nippon Genetics Europe, Cat. No. MG04, Düren, Germany). Sequences of gel-extracted RT-PCR bands were confirmed via Sanger sequencing. Quantification of gel bands was performed using ImageJ software v.1.54g [31].

## 3. Results

### 3.1. Cohort Description

Between 2020 and 2024, a total of 418 families, affected by either NSHL or SHL, were referred to the Medical Genetics Unit of the I.R.C.C.S. “Burlo Garofolo” (Trieste, Italy) and underwent WES analysis. Among these, 195 families (approximately 47%) received a positive molecular diagnosis. Splicing variants accounted for 7% of all HL-related variants detected, with similar proportions in NSHL and SHL cases. Notably, approximately half of these splicing variants are classified as VUS according to the American College of Medical Genetics and Genomics (ACMG) and the Association for Molecular Pathology (AMP) guidelines, highlighting the need for functional studies to clarify their clinical relevance. In this study, we selected seven intronic variants identified in seven families. The variants, none of which had been previously characterized from a functional perspective, were found in the following genes: *ADGRV1*, *ATP11A*, *GSDME*, *OTOF*, *OTOGL*, and *USH2A*. Since in silico predictions suggested a potential impact on splicing, we conducted in vitro splicing assays to functionally validate these predictions and confirm their biological significance. Six out of seven identified variants were found in a heterozygous state, with families A, D, F, and G carrying the identified variants in a compound heterozygous state with another variant in the same gene. Only one variant, the variant c.5104-3C>G within the *OTOF* gene, was in a homozygous state (family E). According to ACMG/AMP guidelines, five out of the seven splicing variants are classified as VUS and two as pathogenic (Table 2).

### 3.2. Family A: ADGRV1 (NM_032119.4) c.9447+1G>A Variant

#### 3.2.1. Clinical and Genetic Evaluation

**Figure 1:**
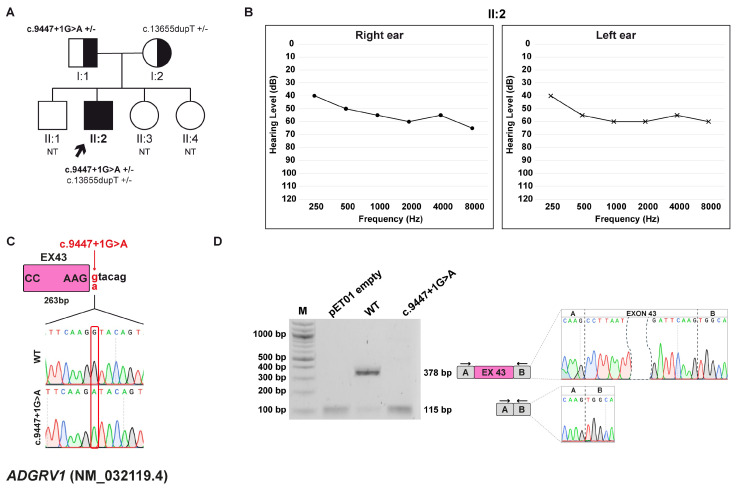
Family A pedigree, auditory phenotype, and splicing effect of the *ADGRV1* (NM_032119.4) c.9447+1G>A variant demonstrated by minigene experiment. (**A**) Pedigree of family A. Squares and circles represent males and female, respectively. The proband (II:2, black arrow) carries the *ADGRV1* c.9447+1G>A variant in compound heterozygous state with the c.13655dupT, p.(Asn4553Glufs*18) variant. Half-filled symbols indicate heterozygous carrier; filled symbols, affected individuals; open symbols, unaffected individuals. Genotypes are reported for each tested individual (+/–, heterozygous for the indicated variant; NT, not tested). (**B**) Pure-tone audiogram of the proband (II:2) showing moderate HL. Air conduction thresholds are indicated as circles (right ear) and crosses (left ear) at each frequency. (**C**) Schematic representation of the *ADGVR1* exon 43 (pink box) and part of the flanking introns subcloned into the pET01 vector, both in the wt and c.9447+1G>A forms. The variant position is highlighted in red. (**D**) RT-PCR analysis with primers spanning pET01 exon A and B (black arrows) after transfection of HEK293T cells with pET01 empty, wt, and c.9447+1G>A vectors. Sequencing chromatograms confirmed the identity of the bands. Exons are separated by dashed lines; a dashed shape separates the initial and terminal regions of the central exon.

The proband, a 10-year-old male, was born to non-consanguineous, healthy parents with no significant family history of genetic disorders or hearing impairment. He was referred for hearing assessments at the age of two due to delayed language development. At that time, he was diagnosed with moderate, non-progressive sensorineural HL and subsequently treated with hearing aids. Moreover, an ophthalmological examination was also performed and yielded normal results. The family pursued genetic counseling to investigate potential hereditary causes and better understand the patient’s condition (Figure 1A,B).

WES analysis identified two compound heterozygous variants within the *ADGRV1* (NM_032119.4) gene, known to be associated with Usher syndrome, type 2C (MIM: #605472). The variants c.13655dupT, p.(Asn4553Glufs*18), and c.9447+1G>A were inherited from the mother and father, respectively. The first variant is reported as pathogenic by the Deafness Variation Database (DVD) and is classified as pathogenic according to the ACMG/AMP criteria. The same variant is described as likely disease-causing in the HGMD [36,37]. The c.9447+1G>A variant, located at the canonical +1 position of 5′ss of exon 43 (Figure 1C), is not described in any public database and is predicted to be pathogenic by the in silico software tools dbscSNV score (ADA/RF) and CADD. Moreover, it was predicted by SpliceAI to disrupt the canonical donor splice site (5′ss) and promote the selection of a cryptic splice site (ss) 3 bp downstream of exon 43.

#### 3.2.2. Minigene Splicing Assay

In vitro splicing assay showed that the wt transcript is 378 bp in length, consistent with correct splicing with inclusion of exon 43, which is 263 bp long. In contrast, the c.9447+1G>A derived transcript is 115 bp long and lacks the entire exon 43. The exon skipping alters the open reading frame (ORF) and leads to the formation of a premature termination codon (PTC) at p.(Ala3062Glyfs*16) (Figure 1D).

### 3.3. Family B: ATP11A (NM_015205.3) c.1221+5G>C Variant

#### 3.3.1. Clinical and Genetic Evaluation

**Figure 2:**
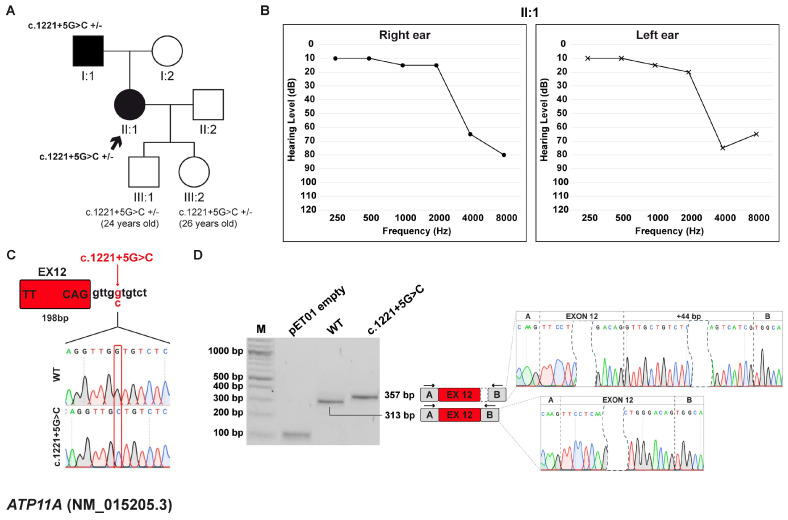
Family B pedigree, auditory phenotype, and splicing effect of the *ATP11A* (NM_015205.3) c.1221+5G>C variant. (**A**) Pedigree of family B. Squares and circles represent males and female, respectively. The proband (II:1, black arrow) carries the *ATP11A* c.1221+5G>C variant in heterozygosis. Filled and open symbols indicate affected and unaffected individuals, respectively. Genotypes are reported (+/–, heterozygous for the indicated variant). (**B**) Pure-tone audiograms of the patient (II:1) reveal moderate-to-severe hearing loss at high frequencies. Air conduction thresholds are indicated as circles (right ear) and crosses (left ear) at each frequency. (**C**) Schematic representation of the *ATP11A* exon 12 (red box) and part of its surrounding introns subcloned into the pET01 vector, both in the wt and c.1221+5G>C forms. The variant position is highlighted in red. (**D**) RT-PCR analysis with primers spanning pET01 exon A and B (black arrows) after transfection of HEK293T cells with pET01 empty, wt, and c.1221+5G>C vectors. Sequencing chromatograms confirmed the identity of the bands. Exons are separated by dashed lines; a dashed shape separates the initial and terminal regions of the central exons.

The proband, a 57-year-old female, underwent her first audiological assessment at the age of 54, which revealed a moderate-to-severe, bilateral, symmetric sensorineural HL, predominantly affecting the high frequencies, particularly at 4000 and 8000 Hz. The patient was born to non-consanguineous parents, both with a history of HL. The mother, aged 83, reports age-related hearing loss (presbycusis), while the father, aged 86, presents with asymmetric HL ranging from mild to profound, with a markedly profound loss in the left ear, predominantly in the high-frequency range. The proband has two children, a 24-year-old son and a 26-year-old daughter, and both reported to be in good health at the time of genetic counseling. Given the resemblance between the patient’s and the father’s audiometric profiles, the family pursued genetic counseling to investigate a potential hereditary underlying the condition (Figure 2A,B).

Trio WES analysis identified the heterozygous c.1221+5G>C variant in the *ATP11A* gene (NM_015205.3), inherited from the father. *ATP11A* is associated with autosomal dominant deafness-84 (DFNA84; MIM #619810), an NSHL form, characterized by a slow progression, typically with onset in the first or second decade of life. Segregation analysis in the offspring of the proband revealed that both children inherited the variant from their mother. This variant is predicted to disrupt a splice donor site and to be deleterious by the in silico prediction software dbscSNV. It is considered potentially pathogenic according to CADD and is not listed in the reference databases HGMD or DVD. Currently, the variant is classified as a VUS according to ACMG/AMP guidelines. Furthermore, the c.1221+5G>C variant, located at position +5 downstream of exon 12 (Figure 2C), was predicted by SpliceAI to determine the loss of the canonical 5′ss and the activation of a cryptic ss.

#### 3.3.2. Minigene Splicing Assay

Minigene splicing assay confirmed in silico prediction: the wt construct generated a transcript of 313 bp corresponding to correct splicing with inclusion of exon 12 (198 bp long). Conversely, the variant induced the selection of a cryptic 5′ss 44 bp downstream of the canonical site, resulting in the formation of a 357 bp transcript with retention of 44 bp of intron 12. This aberrant splicing event causes a change in the ORF and leads to the generation of a PTC at p.(Glu409Glyfs*4) (Figure 2D).

### 3.4. Family C: GSDME (NM_004403.3) c.1183+1G>A Variant

#### 3.4.1. Clinical and Genetic Evaluation

**Figure 3:**
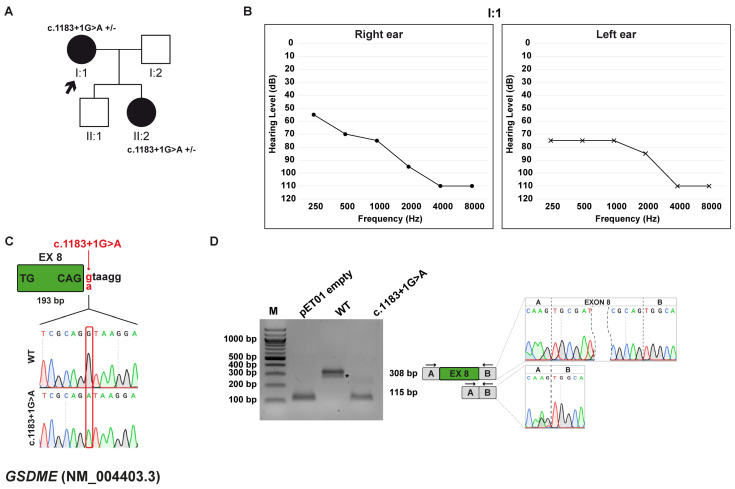
Family C pedigree, auditory phenotype, and splicing effect of the *GSDME* (NM_004403.3) c.1183+1G>A variant demonstrated by minigene experiment. (**A**) Pedigree of family C. Squares and circles represent males and female, respectively. The proband (I:1, black arrow) carries the *GSDME* c.1183+1G>A variant in heterozygosis. Filled and open symbols indicate affected and unaffected individuals, respectively. Genotypes are reported (+/–, heterozygous for the indicated variant). (**B**) Pure-tone audiograms of the patient (I:1) showing severe to profound hearing loss at high frequencies. Air conduction thresholds are indicated as circles (right ear) and crosses (left ear) at each frequency. (**C**) Schematic representation of the *GSDME* exon 8 (green box) and part of its flanking introns subcloned into the pET01 vector, both in the wt and c.1183+1G>A forms. The variant position is highlighted in red. (**D**) RT-PCR analysis with primers spanning pET01 exon A and B (black arrows) after transfection of HEK293T cells with pET01 empty, wt, and c.1183+1G>A vectors. Sequencing chromatograms confirmed the identity of the bands. Exons are separated by dashed lines; a dashed shape separates the initial and terminal regions of the central exons. *, non-specific band.

The proband is a 58-year-old female presenting with isolated, progressive, bilateral, and symmetric sensorineural HL, with onset during childhood. When she was about 28 years old, she started wearing hearing aids to manage her hearing impairment. Currently, cochlear implants are being considered. The patient has two children: one healthy son and a daughter who was diagnosed with bilateral sensorineural HL at the age of 10, with the need to use hearing aids from her teens. The family history, with several other affected first-, second-, and third-degree relatives, both male and female, reveals a pattern of HHL, consistent with an autosomal dominant inheritance (Figure 3A,B).

WES study allowed the discovery of the heterozygous variant c.1183+1G>A within the *GSDME* (NM_004403.3) gene, which is associated with autosomal dominant deafness-5 (DFNA5; MIM: #600994). The variant is predicted to be deleterious by the in silico prediction tools (CADD and dbscSNV) and is reported as likely pathogenic by the DVD. Furthermore, the variant segregated with the HHL phenotype in this family, and is classified as pathogenic according to the ACMG/AMP criteria. The variant is not listed in the HGMD, which, however, reports the c.1183+1G>C and c.1183+1G>T variants affecting the same nucleotide position and classified as deleterious. The pathogenicity of the c.1183+1G>C variant has been demonstrated through functional studies [38]. SpliceAI predicted that the c.1183+1G>A variant, located at the canonical +1 position of 5′ss of exon 8 (Figure 3C), leads to the loss of the 5′ss.

#### 3.4.2. Minigene Splicing Assay

In vitro functional analysis showed that, while the wt minigene generated a 308 bp transcript that includes exon 8, the c.1183+1G>A variant disrupts the 5′ss and induces the complete skipping of exon 8 (Figure 3D). This splicing defect generates a frameshift that introduces a premature termination codon, p.(Cys331Lysfs*42), leading to a truncated protein lacking the normal C-terminal domain. This mechanism is consistent with previously reported gain-of-function variants in *GSDME* clustered around exon 8 [38,39].

### 3.5. Family D: OTOF (NM_001287489.2) c.5533+13G>T Variant

#### 3.5.1. Clinical and Genetic Evaluation

**Figure 4:**
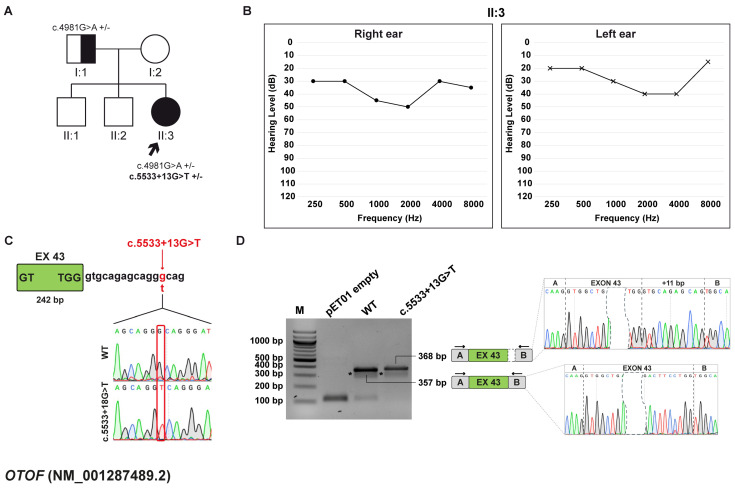
Family D pedigree, auditory phenotype, and splicing effect of the *OTOF* (NM_001287489.2) c.5533+13G>T variant demonstrated by minigene experiment. (**A**) Pedigree of family D. Squares and circles represent males and female, respectively. The patient (II:3, black arrow) carries the c.4981G>A, p.(Glu1661Lys) variant inherited from the father (I:1) and the de novo c.5533+13G>T variant within *OTOF* gene. Half-filled symbols indicate heterozygous carrier; filled symbols indicate affected individuals; open symbols indicate unaffected individuals. Genotypes are reported (+/–, heterozygous for the indicated variant). (**B**) Pure-tone audiograms of the patient (II:3) showing moderate HL. Air conduction thresholds are indicated as circles (right ear) and crosses (left ear) at each frequency. (**C**) Schematic representation of the *OTOF* exon 43 (light green box) and part of its flanking introns subcloned into the pET01 vector, both in the wt and c.5533+13G>T forms. The variant position is highlighted in red. (**D**) RT-PCR analysis with primers spanning pET01 exon A and B (black arrows) after transfection of HEK293T cells with pET01 empty, wt, and c.5533+13G>T vectors. Sequencing chromatograms confirmed the identity of the bands. Exons are separated by dashed lines; a dashed shape separates the initial and terminal regions of the central exons. * non-specific bands.

The proband is a 19-year-old female who underwent a comprehensive audiological examination, which revealed mild sensorineural HL accompanied by central auditory dysfunction consistent with auditory processing disorder. Additionally, the patient reports a reduction in hearing ability following activities that elevate body temperature, such as physical fatigue and exercise, or fever. This pattern of symptom exacerbation suggests a possible sensitivity of the auditory system to physiological stressors. No other significant family history of hearing impairment or related disorder was reported. Given the complexity of the patient’s symptoms, the patient was referred to genetic counseling, and testing was pursued to investigate potential hereditary contributions to her condition (Figure 4A,B).

WES identified two variants in the *OTOF* gene (NM_001287489.2), which are associated with autosomal recessive non-syndromic auditory neuropathy (AUNB1), including temperature-sensitive auditory neuropathy (TS-ANSD), as well as prelingual mild-to-profound sensorineural hearing loss (DFNB9; MIM: #601071) [40]. The first variant, c.4981G>A, p.(Glu1661Lys), is inherited from the father, and the second, c.5533+13G>T, located at position +13 downstream of exon 43 (Figure 4C), occurred de novo. The first variant is reported as disease-causing by HGMD and pathogenic by DVD and ACMG/AMP guidelines. The second variant is a VUS according to DVD and ACMG/AMP guidelines. In addition, the SpliceAI tool forecasted that the c.5533+13G>T variant likely disrupts the canonical 5′ss of exon 43 and leads to the selection of a new one 11 bp downstream of the canonical site.

#### 3.5.2. Minigene Splicing Assay

In vitro assay confirmed SpliceAI prediction, while the wt construct yielded a 357 bp transcript consistent with proper exon 43 inclusion, the one generated by the c.5533+13G>T variant produces a 368 bp transcript, which matches with the selection of a cryptic 5′ss 11 bp after the end of exon 43 (Figure 4D). This splicing alteration disrupts the ORF and causes the generation of a PTC at p.(Ile1847Glufs*9).

### 3.6. Family E: OTOF (NM_001287489.2) c.5104-3C>G Variant

#### 3.6.1. Clinical and Genetic Evaluation

**Figure 5:**
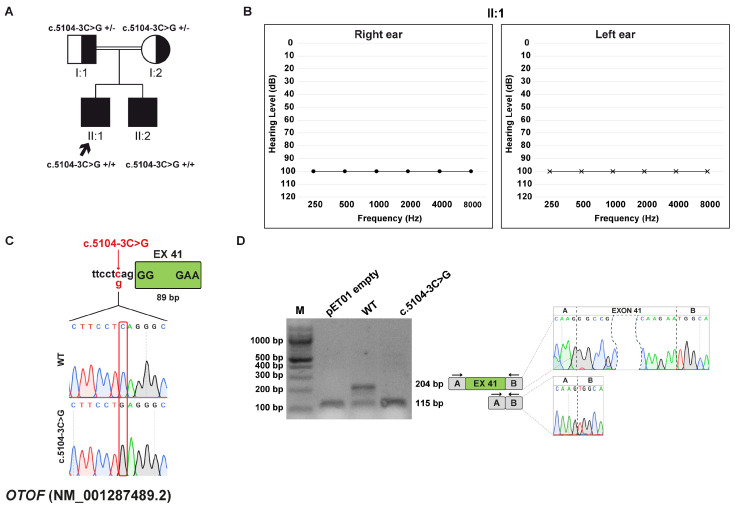
Family E pedigree, auditory phenotype, and splicing effect of the *OTOF* (NM_001287489.2) c.5104-3C>G variant demonstrated by minigene experiment. (**A**) Pedigree of the family E. Squares and circles represent males and female, respectively. The proband (II:1, black arrow) and the patient’s brother (II:2) carry the *OTOF* c.5104-3C>G variant in homozygosity. Half-filled symbols indicate heterozygous carrier; filled symbols indicate affected individuals. For each individual, genotypes are shown as +/– (heterozygous) or +/+ (homozygous) for the indicated variant. (**B**) Pure-tone audiograms of the proband (II:1) revealing bilateral profound HL. Air conduction thresholds are indicated as circles (right ear) and crosses (left ear) at each frequency. (**C**) Schematic representation of the OTOF exon 41 (green box) and part of its flanking introns subcloned into the pET01 vector, both in the wt and c.5104-3C>T forms. The variant position is highlighted in red. (**D**) RT-PCR analysis with primers spanning pET01 exon A and B (black arrows) after transfection of HEK293T cells with pET01 empty, wt, and c.5104-3C>T vectors. Sequencing chromatograms confirmed the identity of the bands. Exons are separated by dashed lines; a dashed shape separates the initial and terminal regions of the central exon.

Two brothers, born to consanguineous healthy parents, presented with normal otoacoustic emissions (OAEs) and altered auditory brainstem response (ABR) during the first year of life, consistent with a diagnosis of auditory neuropathy. The older sibling (II:1), aged 31, was diagnosed with profound HL at three months and received a cochlear implant in the right ear at the age of two. The younger brother (II:2), aged 26, was diagnosed at nine months and underwent cochlear implantation in the left ear at 20 months of age (Figure 5A,B).

WES analysis showed that the two brothers are homozygous for the *OTOF* (NM_001287489.2) c.5104-3C>G variant, inherited from both parents. The variant is predicted to be deleterious by the in silico tools CADD and dbscSNV, and it is classified as VUS according to ACMG/AMP. Notably, DVD lists a different substitution at the same position, c.5104-3C>T, as a VUS. The variant is located 3 bp upstream of exon 41 (Figure 5C) and was predicted to cause the loss of the acceptor splice site by SpliceAI.

#### 3.6.2. Minigene Splicing Assay

Through an in vitro splicing assay, it was observed that the wt transcript is 204 bp long and the transcript harboring the c.5104-3C>T variant is 115 bp, matching the skipping of exon 41, which has an 89 bp length (Figure 5D). The variant alters the ORF and leads to the generation of a PTC at p.(Gly1702Valfs*12).

### 3.7. Family F: OTOGL (NM_173591.7) c.6781+4A>C Variant

#### 3.7.1. Clinical and Genetic Evaluation

**Figure 6:**
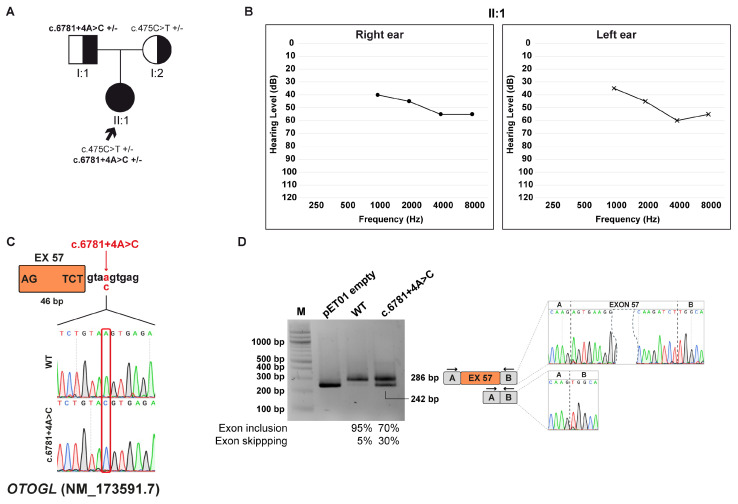
Family F pedigree, auditory phenotype, and splicing effect of the *OTOGL* (NM_173591.7) c.6781+4A>C variant demonstrated by minigene experiment. (**A**) Pedigree of family F. Squares and circles represent males and female, respectively. The proband (II:1, black arrow) carries the *OTOGL* c.6781+4A>C variant, inherited from the father (I:1) and c.475C>T p.(Arg159Trp), inherited from the mother (I:2) in a compound heterozygous state. Half-filled symbols indicate heterozygous carriers; filled symbol indicate affected individual. Genotypes are reported (+/–, heterozygous for the indicated variant). (**B**) Pure-tone audiograms of the patient (II:1) revealing bilateral moderate-severe HL. Air conduction thresholds are indicated as circles (right ear) and crosses (left ear) at each frequency. (**C**) Schematic representation of the *OTOGL* exon 57 (orange box) and part of its flanking introns subcloned into the pET01 vector, both in the wt and c.6781+4A>C forms. The variant position is highlighted in red. (**D**) RT-PCR analysis with primers spanning pET01 exon A and B (black arrows) after transfection of HEK293T cells with pET01 empty, wt, and c.6781+4A>C vectors. Sequencing chromatograms confirmed the identity of the bands. Gel band quantification and the relative frequencies of exon inclusion and skipping are expressed as percentages. Exons are separated by dashed lines; a dashed shape separates the initial and terminal regions of the central exon.

The proband is a 2-year-old girl diagnosed with moderate bilateral sensorineural HL. Specifically, audiological evaluation indicated an ABR threshold of 50 dB in the right ear and 55 dB in the left ear. The patient is the only child of healthy non-consanguineous parents; no additional symptoms were identified during the clinical assessment. Family history is negative for HL or related auditory conditions, suggesting that the patient’s hearing impairment may represent an isolated case (Figure 6A,B).

WES study allowed the discovery of two compound heterozygous variants within the *OTOGL* gene (NM_173591.7), which is associated with autosomal recessive non-syndromic deafness-84B (DFNB84B; MIM: #614944). The first variant, c.6781+4A>C, is inherited from the father, while the second, c.475C>T p. (Arg159Trp) from the mother. The first variant has not been reported in the literature, and both variants are classified as VUS by ACMG/AMP and DVD. Moreover, the c.6781+4A>C variant, located 4 bp downstream exon 57 (Figure 6C) according to SpliceAI, was predicted to disrupt the donor splice site.

#### 3.7.2. Minigene Splicing Assay

Functional validation using minigene splicing assay revealed that both wt and c.6781+4A>C derived transcripts undergo normal splicing with inclusion of exon 57 and a length of 286 bp, as well as exon skipping. Quantification of gel bands with ImageJ showed that these events happen with different frequencies between the wt transcript and the one originating from the variant. In particular, normal splicing accounts for approximately 95% of the transcript produced by the wt minigene and 70% by the c.6781+4A>C minigene. Conversely, exon skipping occurs in the remaining 5% and 30% for wt and c.6781+4A>C products, respectively (Figure 6D). The skipping of the exon 57 causes an alteration of the ORF and leads to the formation of a PTC at p.(Asn2246Alafs*11).

### 3.8. Family G: USH2A (NM_206933.4) c.1551-5T>G variant

#### 3.8.1. Clinical and Genetic Evaluation

**Figure 7:**
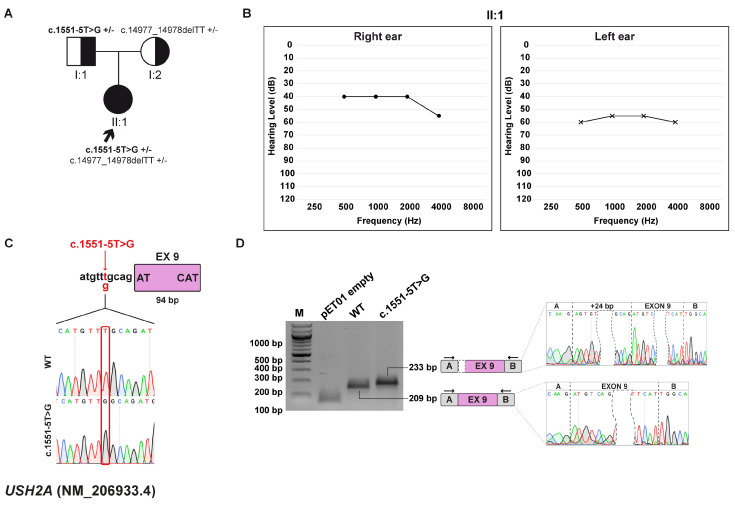
Family G pedigree, auditory phenotype, and splicing effect of the *USH2A* c.1551-5T>G variant demonstrated by minigene experiment. (**A**) Pedigree of family G. Squares and circles represent males and female, respectively. The proband (II:1, black arrow) carries the *USH2A* c.1551-5T>G variant, inherited from the father (I:1) and the c.14977_14978delTT, p.(Phe4993Profs*7), inherited from the mother (I:2) in a compound heterozygous state. Half-filled symbols indicate heterozygous carriers; filled symbol indicate affected individual. Genotypes are reported for each individual (+/–, heterozygous for the indicated variant). (**B**) Pure-tone audiograms of the patient (II:1) reveal bilateral moderate-severe HL. Air conduction thresholds are indicated as circles (right ear) and crosses (left ear) at each frequency. (**C**) Schematic representation of the *USH2A* exon 9 (pink box) and part of its flanking introns subcloned into the pET01 vector both in the wt and c.1551-5T>G forms. The variant position is highlighted in red. (**D**) RT-PCR analysis with primers spanning pET01 exon A and B (black arrows) after transfection of HEK293T cells with pET01 empty, wt, and c.1551-5T>G vectors. Sequencing chromatograms confirmed the identity of the bands. Exons are separated by dashed lines; a dashed shape separates the initial and terminal regions of the central exon.

The patient is a 6-year-old female who was diagnosed with moderate HL at 4 months of age, based on an abnormal ABR test. The patient is the only child of non-consanguineous, healthy parents (Figure 7A,B).

Exome sequencing allowed the identification of two variants within the *USH2A* (NM_206933.4) gene, which is linked to Usher syndrome type 2A (MIM: #276901). The first variant, c.14977_14978delTT, p.(Phe4993Profs*7), is inherited from the mother and leads to the formation of a truncated protein. The variant is predicted to be pathogenic by CADD. It is reported as disease-causing in the HGMD, DVD, and according to ACMG/AMP guidelines. The other variant, c.1551-5T>G, is inherited from the father and is located at a non-canonical position near the acceptor splice site (Figure 7C). While CADD and dbscSNV classify it as not damaging, SpliceAI assigns a delta score indicative of loss of the canonical acceptor site and predicts activation of a cryptic splice site within intron 8. This variant is reported as VUS in the HGMD, where it has been associated with retinitis pigmentosa and retinal diseases [41,42]. Consistently, both the DVD and ACMG/AMP criteria also classify it as a VUS.

#### 3.8.2. Minigene Splicing Assay

Through an in vitro splicing assay, it was observed that the wt transcript and the one produced from the c.1551-5T>G variant are 209 bp and 233 bp long, respectively. The variant, in fact, causes the selection of a cryptic 3′ss 24 bp upstream of the canonical one (Figure 7D). This splicing event results in an in-frame insertion of eight amino acids at the protein level, p.(Arg517_Cys518insValCysAspAlaHisValCysArg), which may alter protein structure or function.

## 4. Discussion

Between 2020 and 2024, 418 families underwent genetic counseling for the evaluation of suspected HHL. In this cohort, a molecular diagnosis was achieved in approximately 47% of cases, consistent with results from other studies [43,44]. Among all HL-related variants identified through WES, splicing variants account for nearly 7% of both NSHL and SHL cases, and about 50% of them are classified as VUS according to ACMG/AMP guidelines. In this study, we focused on a subset of splicing variants, and we employed minigene splicing assays to assess the functional impact of these variants and improve variant interpretation.

In five out of seven cases, the identified variants were located at the 5′ss, either at canonical or non-canonical positions, leading to aberrant splicing events. Among these, exon skipping was observed for variants in *ADGRV1*, *GSDME*, and *OTOGL* genes, while selection of a cryptic splice site that causes partial intron retention was seen in *ATP11A* and *OTOF* (c.5533+12G>T) cases. In four cases, the alternative transcripts introduced a frameshift, generating PTC-containing transcripts, which are typically targeted for degradation by the NMD pathway, thereby preventing the accumulation of truncated, likely nonfunctional proteins [45]. In contrast, the *GSDME* variant produced a truncated protein. Notably, both the present variant and previously reported pathogenic variants cluster around exon 8, underscoring this region as a functional hotspot. These findings indicate that the novel c.1183+1G>A variant in *GSDME* shares the same pathogenic mechanism as other DFNA5-associated variants, inducing exon 8 skipping and producing a truncated protein with toxic gain-of-function effects. This expands the mutational spectrum of *GSDME*-related hearing loss and reinforces the role of exon 8 as a hotspot for pathogenic variants [38,39,46]. The other two variants (*OTOF*, c.5104-3C>G and *USH2A*, c.1551-5T>G) affected the recognition of the canonical 3′ss. The *OTOF* variant led to exon skipping and the production of a PTC-transcript, while the *USH2A* variant resulted in an in-frame insertion, which may alter the structure and function of the resulting protein, warranting further investigation to assess its impact on protein expression and activity.

The functional evidence given by minigene splicing assays provided strong support for the predicted damaging effects of the HL-related variants identified in our cohort, significantly enhancing diagnostic accuracy and contributing to more precise genotype-phenotype correlations. Most patients were diagnosed in early childhood, and identifying the molecular cause of their HL played a key role in defining follow-up and informing parents about reproductive decisions, including the option of preimplantation and prenatal genetic testing.

Our insights may serve as a prognostic tool, which is crucial for SHL forms. For example, two patients carried biallelic variants within the *ADGRV1* and *USH2A* genes, both associated with Usher syndrome. Although these young patients presented with isolated HL, functional assessment of the splicing variant found in compound heterozygosity with previously reported pathogenic variants highlighted the necessity for ongoing clinical surveillance. In particular, regular ophthalmological evaluations are essential due to the elevated risk of developing retinitis pigmentosa. Even in NSHL cases, our findings contributed to guiding clinical management. For example, the children of the patient carrying a heterozygous splicing variant in *ATP11A* were found to carry the same variant, despite not yet showing HL. As a result, periodic audiological evaluations were recommended to monitor for the potential onset of hearing impairment.

Furthermore, our findings may have an impact on therapeutic outcomes. Currently, cochlear implantation (CI) represents the standard treatment for severe-to-profound hearing loss. However, this approach has significant limitations related to the invasiveness of the surgical procedure, patient compliance, and variable efficacy. Patients treated with CI may experience loss of residual hearing and may still exhibit hearing difficulties despite implantation [47]. Moreover, the success of CI is influenced by the underlying etiology of HL, with poorer outcomes typically observed in auditory neuropathies, especially in cases involving postsynaptic dysfunction [48,49].

Since in some cases CI cannot be fully effective [50], gene therapy has recently emerged as a promising alternative approach to treat *OTOF*-related HL. To date, six clinical trials are ongoing (ClinicalTrials.gov ID: NCT06696456, NCT06370351, NCT05901480, NCT05821959, NCT05788536, NCT06722170) and they are all based on rescuing otoferlin function by the direct injection into the cochlea of adeno-associated (AAV)-mediated *OTOF* gene (AAV-*OTOF*). The basic eligibility criteria for study enrollment require participants to have biallelic pathogenic variants in *OTOF*, early-onset severe-to-profound HL, and no prior cochlear implantation. Therefore, precise molecular diagnosis, including functional validation of VUS, is critical for identifying patients eligible for such experimental treatments.

Despite minigenes being a powerful tool to investigate the functional impact of splicing variants, they only partially recapitulate endogenous splicing patterns and do not fully reflect the complexity of cellular regulatory mechanisms. Outcomes can be influenced by the transfection cell line, given differences in splicing factor expression and tissue-specific regulation, as well as by the size and composition of the cloned fragment, as limited inclusion of intronic sequences may exclude essential *cis*-regulatory elements or alter exon–intron context. Consequently, results obtained from minigene assays should be interpreted with caution and ideally validated using patient-derived RNA. Nevertheless, they remain a valuable approach to support variant classification, especially in contexts where in vivo studies are not feasible, such as in HL.

In summary, we identified and functionally validated seven splicing variants associated with HL in the *ADGRV1*, *ATP11A*, *GSDME*, *OTOF*, *OTOGL*, and *USH2A* genes. This study shed light on the importance of addressing splicing variants, often overlooked due to their uncertain significance, through functional validation. These findings emphasize the utility of integrating functional assays into variant interpretation workflow and present significant implications for genetic counseling, from both a diagnostic, prognostic, as well as therapeutic point of view.

## 5. Conclusions

Our study underscores the importance of integrating comprehensive clinical assessment and WES analysis with functional validation of splicing variants in HHL patients. This combined approach allows the assessment of variant effects on splicing, facilitating their reclassification. Functional characterization not only deepens our understanding of their biological role but also improves diagnostic accuracy. Given the clinical and genetic heterogeneity of HL, the minigene splicing assay emerges as an additional tool to help clinicians in providing accurate recurrence risks and prognostic information to patients and their families. Furthermore, functional validation can inform genetic eligibility for clinical trials, broadening patients’ access to experimental treatments such as gene therapy. Collectively, these advances contribute to improved diagnosis and pave the way for more personalized, effective care for individuals affected by HHL.

## Figures and Tables

**Table 1:** List of primers used in the study. Primers were used for gDNA amplification, RT-PCR of cDNAs, and sequencing of splicing products. Sequences homologous to the linearized vector ends are in bold, with the XhoI and NotI restriction sites underlined.

Name	Sequence 5′- -3′	Purpose
*ADGRV1*-gDNA_F	**CGGGCCCCCCCTCGA**CATCAGGTTAAGGAAGCTGCCT	Amplification for cloning
*ADGRV1*-gDNA_R	**ACCGCGGTGGCGGCC**CCAGTTGCGCTTTATACAGAGT	
*ATP11A*-gDNA_F	**CGGGCCCCCCCTCGA**CCGCCCATAGTCCTTGTC	Amplification for cloning
*ATP11A*-gDNA_R	**ACCGCGGTGGCGGCC**TGTAAATGTAACAGCACGGC	
*GSDME*-gDNA-F	**CGGGCCCCCCCTCGA**GCCAGGTTCAGCTTACTGTCCC	Amplification for cloning
*GSDME*-gDNA-R	**ACCGCGGTGGCGGCC**CCGAAGGGGGGTTTCCCATC	
*OTOF (c.5533+13G>T)*-gDNA-F	**CGGGCCCCCCCTCGA**GCCCACAGACAAGCATGTGGC	Amplification for cloning
*OTOF (c.5533+13G>T)*-gDNA-R	**ACCGCGGTGGCGGCC**GCCACATGCTTGTCTGTGGGC	
*OTOF (c.5104-3C>G)*-gDNA-F	**CGGGCCCCCCCTCGA**GCTCAGGCAGAGACCTGGG	Amplification for cloning
*OTOF (c.5104-3C>G)*-gDNA-R	**ACCGCGGTGGCGGCC**CTCTCCAGGGCAGGCCCTAAAC	
*OTOGL*-gDNA-F	**CGGGCCCCCCCTCGA**TAATGTAATTTTAAACAAAATATCTACTCC	Amplification for cloning
*OTOGL*-gDNA-R	**ACCGCGGTGGCGGCC**ATACAAAAACCTTTAAGTTAAAGAC	
*USH2A*-gDNA-F	**CGGGCCCCCCCTCGA**CCACACGATTAAGGAGAAACTG	Amplification for cloning
*USH2A*-gDNA-R	**ACCGCGGTGGCGGCC**TCAGTTGTAATTTCTCTGGCTG	
pET01-EX1_RT-PCR_F	GATCGATCCGCTTCCTGCCCC	pET01 cDNA amplification
pET01-EX2_RT-PCR_R	CTGCCGGGCCACCTCCAGTGCC	
pET01-EX1_SEQ_F	GGATTCTTCTACACACCC	pET01 sequencing primers
pET01-EX2_SEQ_R	TCCACCCAGCTCCAGTTG	

**Table 2:** Summary of the splicing variants identified in this study. The table provides an overview of the splicing variants detected in this study, including the corresponding family ID, gene, transcript, and variant. Information on inheritance pattern and zygosity is reported for each case. Variant classifications are based on ACMG/AMP guidelines and Varsome [32] with specific criteria and strength indicated in brackets [33,34]. Annotations from the Deafness Variation Database (DVD) [35] are also included. Predictive scores for splicing impact are provided: CADD, dbscSNV (ADA and RF scores; ADA: AdaBoost; RF: Random Forest), and SpliceAI. For SpliceAI, predicted splicing effects are reported with their corresponding delta scores, including DL (donor loss), DG (donor gain), AL (acceptor loss), and AG (acceptor gain). NA: not available.

Family ID	Gene (Transcript)	Inheritance	Zigosity	Variant	ACMG/AMP	DVD	CADD	dbscSNV Score (ADA/RF)	SpliceAI Delta Score
**A**	*ADGRV1* (NM_032119.4)	AR	Compound Heterozygous	c.9447+1G>A	Path (PVS1, PM2_Supp, PM3)	NA	33	0.999/0.938	DL 0.98, DG 0.53
**B**	*ATP11A* (NM_015205.3)	AD	Heterozygous	c.1221+5G>C	VUS (PM2_Supp, PP3, PP1)	NA	23.3	0.999/0.982	DL 0.97, DG 0.62
**C**	*GSDME* (NM_004403.3)	AD	Heterozygous	c.1183+1G>A	Path (PVS1, PM2_Supp, PP1_Supp)	Likely Path	33	0.999/0.814	DL 1.00, DG 0.78
**D**	*OTOF* (NM_001287489.2)	AR	Compound Heterozygous	c.5533+13G>T	VUS (PM2_Supp)	VUS	4.6	NA	DL 0.84, DG 1.00
**E**	*OTOF* (NM_001287489.2)	AR	Homozygous	c.5104-3C>G	VUS (PM2_Supp, PP3, PM3_Supp)	NA	24.4	0.999/0.96	AL 0.96, AG 0.32
**F**	*OTOGL* (NM_173591.7)	AR	Compound Heterozygous	c.6781+4A>C	VUS (PM2_Supp, PP3)	VUS	22.7	0.999/0.916	DL 0.21
**G**	*USH2A* (NM_206933.4)	AR	Compound Heterozygous	c.1551-5T>G	VUS (PM2_Supp, PM3)	VUS	17.5	0.358/0.486	AL 0.50, AG 0.25

## Data Availability

WES datasets have not been deposited in a public repository because of privacy and ethical restrictions.

## Abbreviations

The following abbreviations are used in this manuscript:

HL	Hearing loss
HHL	Hereditary hearing loss
WES	Whole-exome sequencing
SHL	Syndromic hearing loss
NSHL	Non-syndromic hearing loss
AD	Autosomal dominant
AR	Autosomal recessive
XL	X-linked
NGS	Next-generation sequencing
VUS	Variant of unknown significance
HGMD	Human Gene Mutation Database
CADD	Combined Annotation Dependent Depletion
gDNA	Genomic DNA
wt	Wild-type
ACMG	American College of Medical Genetics and Genomics
AMP	Association for Molecular Pathology
DVD	Deafness Variation Database
ORF	Open reading frame
PTC	Premature termination codon
ss	Splice site
OAEs	Otoacoustic emissions
ABR	Auditory brainstem response
CI	Cochlear implantation
